# Transcriptional Riboswitches Integrate Timescales for Bacterial Gene Expression Control

**DOI:** 10.3389/fmolb.2020.607158

**Published:** 2021-01-13

**Authors:** Catherine E. Scull, Shiba S. Dandpat, Rosa A. Romero, Nils G. Walter

**Affiliations:** Department of Chemistry, Single Molecule Analysis Group and Center for RNA Biomedicine, University of Michigan, Ann Arbor, MI, United States

**Keywords:** RNA polymerase, RNA folding, riboswitch control of gene expression, transcription, structural dynamics

## Abstract

Transcriptional riboswitches involve RNA aptamers that are typically found in the 5′ untranslated regions (UTRs) of bacterial mRNAs and form alternative secondary structures upon binding to cognate ligands. Alteration of the riboswitch's secondary structure results in perturbations of an adjacent expression platform that controls transcription elongation and termination, thus turning downstream gene expression “on” or “off.” Riboswitch ligands are typically small metabolites, divalent cations, anions, signaling molecules, or other RNAs, and can be part of larger signaling cascades. The interconnectedness of ligand binding, RNA folding, RNA transcription, and gene expression empowers riboswitches to integrate cellular processes and environmental conditions across multiple timescales. For a successful response to an environmental cue that may determine a bacterium's chance of survival, a coordinated coupling of timescales from microseconds to minutes must be achieved. This review focuses on recent advances in our understanding of how riboswitches affect such critical gene expression control across time.

## Introduction

Over the past several decades, RNA has emerged as a key player beyond a “message” between DNA and protein. Non-coding RNAs (ncRNAs) are vital for countless cellular mechanisms, such as ribozyme mediated catalysis of RNA processing reactions, RNA mediated gene silencing, and stabilization of phase separated particles, to name a few (Eddy, 2001; Walter and Engelke, 2002; Eulalio et al., 2007; Serganov and Patel, 2007; Carthew and Sontheimer, 2009; Palazzo and Lee, 2015; Ravikumar et al., 2019; Herviou et al., 2020; Tollerson and Ibba, 2020). The functionality of RNA can be attributed to its propensity to fold into a variety of different structures on a rugged free-energy landscape (Chen and Dill, 2000; Thirumalai et al., 2001; Mustoe et al., 2014). To form functional, dynamic structures, RNA must overcome internal electrostatic repulsion of its phosphate backbone to form stable hydrogen-bonding between heteroatoms in the form of both Watson-Crick and non-Watson-Crick base pairs and sugar-base interactions (Rich, 2009). In the cell, these structural and conformational transitions occur in the presence of counterions, metabolites, small molecules, and proteins that form a plethora of interactions with functional RNAs to achieve critical cellular outcomes (Winkler et al., 2003; Mandal and Breaker, 2004; Breaker, 2012; Frieda and Block, 2012; Suddala et al., 2019; Chauvier et al., 2020; Zhang, 2020). A complete study of the structural dynamics of RNA is crucial for understanding its role beyond its canonical function as a coding messenger RNA (mRNA). Emerging biophysical techniques such as single-molecule microscopy, multi-dimensional NMR, and most recently, near-atomic resolution cryo-electron microscopy (cryo-EM) are yielding new insights into the time-dependent evolution of RNA structures (Tinoco et al., 2010; Ray et al., 2019; Chang et al., 2020).

**Figure 1:**
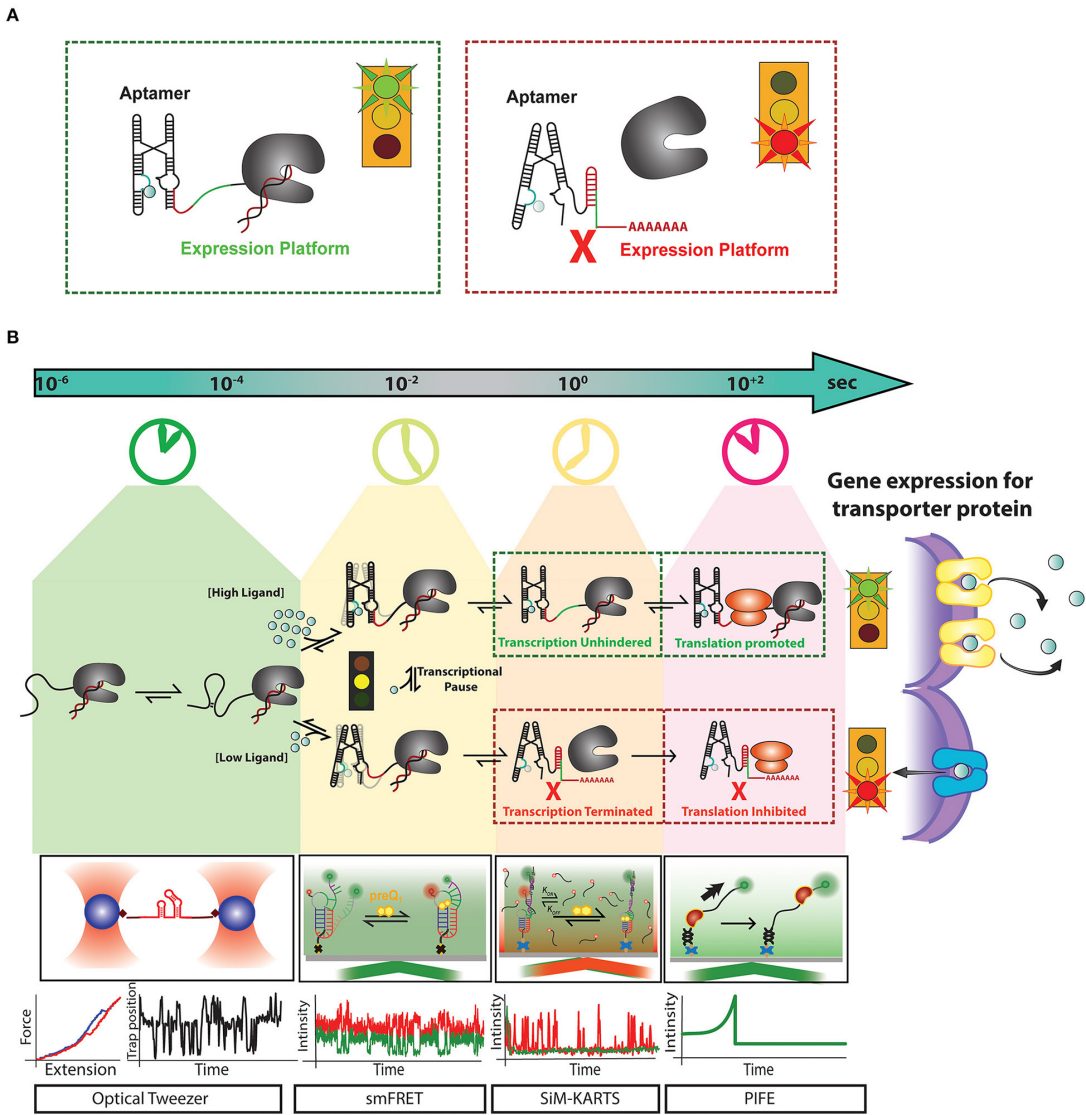
Recent advances in single-molecule techniques have allowed for the study of transcriptional riboswitches on a variety of biologically relevant timescales: **(A)** Transcriptional riboswitches, such as the Mn^2+^ riboswitch, consist of a ligand binding, or “aptamer” region (cyan) that controls the shape of the “expression platform” region (red) to ultimately control transcription termination by the transcribing RNAP (gray). **(B)** Riboswitch mediated upregulation of a gene happens co-transcriptionally where events like RNA folding leading to aptamer formation occur at a timescale of micro-second to milliseconds. After partial aptamer formation, ligand binding events compete with tertiary folding events which happen in the timescale of milliseconds to seconds. Events such as transcriptional pausing play an essential role in regulating the ligand binding and RNA folding events at the co-transcriptional level. For a riboswitch upregulating gene expression for metal ion transporter proteins, at high concentration metal ion, the riboswitch binds them as ligand and from anti-terminator promoting transcription and releases ribosome binding site available for initiating translation. The formation of transporter proteins due to this signaling releases excess metal ions out of cytoplasm to avoid toxicity. In contrast, when there is a low concentration of metal ions required for cell function, riboswitch for terminator hairpin to prevent transcription and sequesters ribosome binding site to block translation. Single-molecule techniques like optical tweezer have been very effective in measuring events at a faster timescale to monitor stepwise RNA folding and unfolding. smFRET has been adapted as a useful tool to monitor the interaction between RNA folding and ligand binding. Single-molecule methods like Single-Molecule Kinetic Analysis of RNA Transient Structures (SiM-KARTS) been used as an essential tool to probe changes in RNA structure as an alternative to SMFRET that requires site-specific labeling and can be extended to study binding events happening during co-transcriptional events. Protein induced fluorescence enhancement (PIFE) has emerged as an important tool to monitor the speed of transcription and activity of RNA polymerase happening in the timescale of seconds. These unique techniques have been essential for the study of riboswitch regulated events at different timescales. However, a combination of one or more of these techniques will be a powerful tool to decipher the real-time mechanism of the expressome at each step of gene regulation.

Riboswitches are a group of dynamic ncRNA motifs that exist almost exclusively in prokaryotes (Blount and Breaker, 2006), although there have been a few riboswitches described in eukaryotes that appear to modulate splicing (McCown et al., 2017), and more recently some riboswitch-like elements were discovered in viral genomes (Chahal et al., 2019). Riboswitches are usually found upstream, in the 5′ untranslated region of mRNAs, where they regulate transcription and translation through binding of their cognate ligand to their aptamer domain (Figure 1A). Aptamers have evolved to bind diverse ligands, from small molecules (often metabolites like s-adenosyl-methionine and preQ_1_), to cations (such as Mn^2+^), anions (such as F^−^), and even other RNAs (such as tRNAs) (Poiata et al., 2009; Suddala et al., 2015; Widom et al., 2018; Chauvier et al., 2019; Zhang, 2020). Upon binding to their cognate ligands, riboswitches alter the secondary structure of a downstream domain, termed the expression platform, which turns “on” or “off” either transcription termination or translation initiation (Figure 1A, Widom et al., 2018). This review primarily focuses on the function of riboswitches involved in transcription regulation, encompassing events on timescales ranging from ligand binding to RNA folding, RNA transcription, and far-reaching cellular gene expression control.

Transcriptional riboswitch activity can essentially be broken down into 4 steps that occur on distinct timescales: (1) ligand influx, which can be as fast as the rate of diffusion, (2) ligand binding to the RNA, establishing specific hydrogen bonding, stacking and ionic interactions (nanoseconds to single seconds), (3) alterations in RNA secondary structure (milliseconds to multiple seconds), and (4) regulation of transcription elongation/termination and their downstream biological consequences (seconds to minutes) (Figure 1B) (Al-Hashimi and Walter, 2008). Until recently, studies of riboswitch folding were often executed in the absence of the transcriptional machinery (Duesterberg et al., 2015), despite the reality that riboswitch folding *in vivo* occurs co-transcriptionally (Frieda and Block, 2012). This leaves a gap between the field's understanding of riboswitch activity from molecular to cellular levels. RNA structures have recently been revealed to impact active site conformations and transcription activity of bacterial RNA polymerase (RNAP) in both *cis* and *trans* (Sedlyarova et al., 2017; Kang et al., 2018). A study by the Walter lab unveiled that the nascent preQ_1_ riboswitch's secondary structure directly influences pausing behavior of the transcribing RNAP (Widom et al., 2018). This study illustrates that both the template DNA and RNAP have a significant impact on riboswitch folding, and vice versa. Thus, to achieve proper control of transcript synthesis and ultimately protein expression, the four steps of transcriptional riboswitch activity must be kinetically coupled (Ray et al., 2019).

## Coordination of Riboswitch Activity for Advantageous Biological Outcomes

The timescales associated with gene regulation vary widely in both bacteria and eukaryotes, and are dependent on fine-tuned cellular sensitivity to external environmental signals (Hargrove et al., 1991; Shamir et al., 2016). Due to the complexity and compartmentalization of eukaryotic cells, gene regulation is relatively isolated, both spatially and temporally (Mandal and Breaker, 2004; Ralston, 2008). By contrast, in simpler organisms such as bacteria, with generally little membrane-enclosed sub-cellular compartmentalization, the colocalization of transcription and translation of their genes (Ralston, 2008) engenders regulation through direct coupling of processes. This makes bacteria a simple yet elegant model to study gene regulation (Proshkin et al., 2010; Kohler et al., 2017). Leveraging this coupled system, bacteria have evolved a variety of motifs within the nascent mRNA, including riboswitches and specific sequence elements, that induce transcriptional pausing and backtracking (Zhang et al., 2010; Perdrizet et al., 2012; Steinert et al., 2017). Furthermore, *Escherichia coli* (*E. coli*) bacteria have even been shown to directly couple transcription and translation, with ribosomes binding to mRNA during active transcription elongation by RNAP (Kohler et al., 2017; O'Reilly et al., 2020). This feature allows for the precise orchestration of gene regulation through the formation of the tightly coupled and highly efficient machinery termed the “expressome” (Proshkin et al., 2010; Kohler et al., 2017; O'Reilly et al., 2020; Washburn et al., 2020). The signal of a small ligand affecting the local structure of a riboswitch can then be transduced into a profound change in expressome function through a wave of kinetic selection, creating a system analogous to the struggle of “David vs. Goliath,” where a tiny metabolic ligand has the ability to control the activity of the giant expressome (Ray et al., 2019).

**Figure 2:**
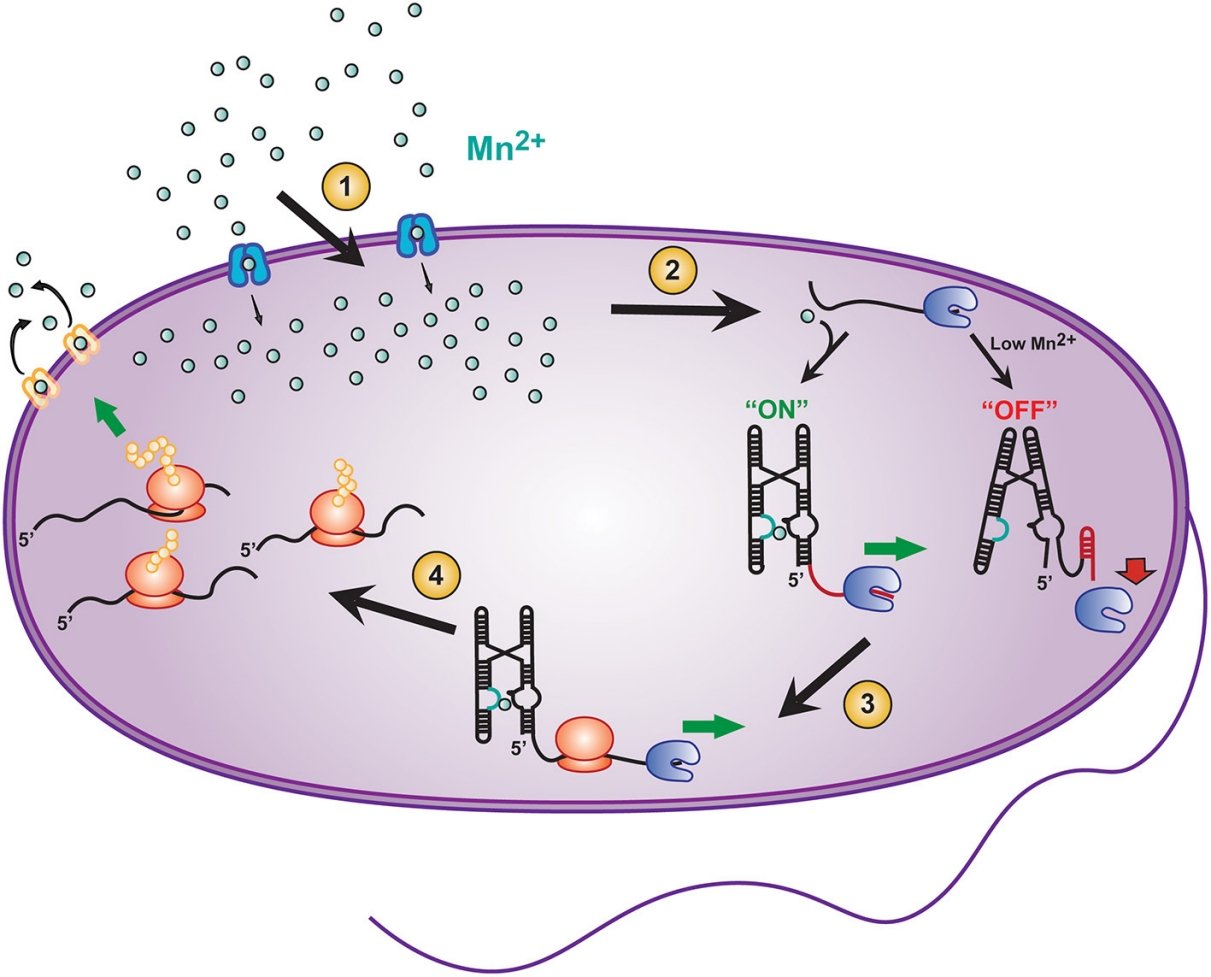
Transcriptional riboswitches, such as the Mn^2+^ riboswitch, involve integration of various time scales to modulate gene expression and maintain cellular homeostasis in *E. coli*. A number of coordinated steps are required for riboswitch mediated maintenance of cellular Mn^2+^ homeostasis: (1) Modulation of Mn^2+^ transport into the cell by mntH (*blue transporter*), (2) binding of Mn^2+^ to the *yybP-ykoY* riboswitch on the 5′ UTR of the *mntP* gene to permit transcription elongation by RNAP (*blue packman*), (3) co-transcriptional translation of *mntP* to ultimately result in, (4) increased expression of the mntP transporter (*yellow transporter*) which exports excessive intracellular Mn^2+^.

The connection of fast intermolecular reactions (ligand influx and binding) to relatively slow global gene regulation is a critical modulator for sustaining the life of prokaryotes. For example, the Mn^2+^ sensing riboswitch found in *Salmonella* modulates the uptake of the transition metal ion Mn^2+^, which is required for the virulence of this pathogenic bacterium (Shi et al., 2014). *Salmonella* is an intracellular pathogen that is phagocytized by host immune cells and resides in specialized cellular compartments known as *Salmonella*-containing vacuoles (SCVs) (Zaharik et al., 2004). SCVs contain host transmembrane transporters (such as Nramp1) that remove divalent cations from the vacuole to starve the pathogen's supply of essential cofactors (Forbes and Gros, 2001; Shi et al., 2014). For this reason, once internalized by SCVs, *Salmonella* must carefully balance intracellular concentrations of divalent cations to maintain sufficient but permissive concentration of Mn^2+^ ions. (Forbes and Gros, 2001; Shi et al., 2014). This balance is mediated in part by a Mn^2+^ sensitive riboswitch found upstream of a gene coding for mntH, a Mn^2+^ specific transporter (Figure 2, blue Mn^2+^ transport protein). When *Salmonella* is deficient in Mn^2+^, transcription elongation of the mntH gene becomes permissive, subsequently allowing for expression of the transporter and resulting in an increase in Mn^2+^ uptake (Shi et al., 2014). Once sufficient Mn^2+^ has been imported, binding of excess Mn^2+^ to the Mn^2+^ riboswitch turns “off” expression of mntH so that concentration levels of the divalent cation do not result in cytotoxicity (Forbes and Gros, 2001; Shi et al., 2014).

In addition to the mntH gene, *E. coli* contains an efflux transporter protein, called mntP (Figure 2, yellow Mn^2+^ transport protein) (Waters et al., 2011). mntP's expression can be controlled at both the transcriptional and translational levels by the *yybP–ykoY* Mn^2+^ riboswitch (Suddala et al., 2019), which induces both transcription elongation and translation of *mntP* upon binding Mn^2+^ ions at a sub-cytotoxic concentration. After expression, mntP exports excess divalent cation when its concentration exceeds permissive levels (Dambach et al., 2015). Clearly, mediation of genetic control by riboswitches is both highly dynamic and critical for maintaining a proper balance of Mn^2+^ homeostasis in bacteria.

## Toward a Holistic Understanding of Riboswitch Function

While biochemical and genetic studies have been essential for identification of functional riboswitches, critical advances in studies *in vivo* as well as *in vitro* have allowed us to understand how small molecular interactions in riboswitches ultimately propagate into global changes in gene expression. A recently developed technique called *Re*porter *Co*upled *I*n *C*ell *S*elective 2′-*H*ydroxyl *A*cylation analyzed by *P*rimer *E*xtension (ReCo-icSHAPE) is one approach by which the coupling of ligand influx to ligand-induced folding of a translational riboswitch and the subsequent impact on the expression of a reporter gene can be monitored directly (Dutta et al., 2018). Using this strategy, a preQ_1_ type II translational riboswitch from *Lactobacillus rhamnosus* (Lrh) was probed in *E. coli*, confirming and complementing expression studies of a GFP-coupled reporter. Through intracellular icSHAPE, this study revealed stronger preQ_1_ mediated occlusion of the ribosome binding site than was observed in the previously described structure, which was solved in purified form via X-ray deffraction (Dutta et al., 2018). Although this approach was used here to probe a translational riboswitch, in the future, this tool may be utilized for directly monitoring the coordination of poorly understood transcriptional riboswitches. This would allow for holistic studies of all stages of riboswitch mediated gene control from the initiation of transcription to ultimate protein expression (Figure 1B).

### Riboswitches in Prokaryotes Allow for Tight Coupling of Ligand Influx and Gene Expression

In the precisely organized gene regulatory systems of bacteria, the efficiency of riboswitch regulation is dependent on the speed of transcription, long before the fate of translation is decided (Wickiser et al., 2005; Garst and Batey, 2009). That is, folding of the nascent RNA transcript in the wake of the elongating RNAP, and in response to ligand binding, will determine the outcome of gene expression (Figure 2). Due to such direct coupling of RNA folding with transcription, riboswitches are considered to kinetically control the regulation of the downstream genes (Wickiser et al., 2005). In the sequence of events, the binding rate of the ligand, and the folding rate of the RNA may be faster (nanosecond to millisecond timescales) than the rate of transcription (milliseconds to seconds) (Wickiser et al., 2005; Gilbert et al., 2006; Roberts et al., 2008; Breaker, 2012; Watters et al., 2016). Hence the cellular concentration of ligand must be higher than its dissociation constant (K_D_) to allow for the ligand binding kinetics to outpace and thus drive the kinetics of RNA folding. A commonly observed mechanism called the induced fit (IF) mechanism describes riboswitch ligand binding events where ligand binding occurs faster than the conformational changes of the riboswitch, whereas ligand dissociation from the unfolded RNA is slower (Gilbert et al., 2006). Alternatively, the conformation selection (CS) model describes riboswitches where the RNA conformational change occurs faster than ligand binding, and ligand dissociates too rapidly from the unfolded RNA to achieve the IF mechanism (Suddala et al., 2015). The transition between the IF and CS models is governed, on one hand, by the ligand concentration and, on the other hand, by temperature and cofactors affecting RNA folding such as the cationic micro-environment. Undoubtedly, evolutionary pressures shape the sequence composition of the riboswitch to finetune this balance to the cell's needs (Suddala and Walter, 2014; Suddala et al., 2015; Rode et al., 2018). Ligand recognition mechanisms like the CS and IF models have provided the basis for the kinetic selection of transcriptional riboswitches (Suddala and Walter, 2014).

For some of the best described riboswitches, it is thought that the IF model is the prevailing mechanism driving riboswitch folding. However, aside from the challenges to accurately distinguish the two mechanisms, there exist examples of riboswitches where it is thought that (subtle) structural rearrangement occurs faster than ligand binding, hence gene regulation occurs via CS (Suddala et al., 2015). One example of such a finely tuned riboswitch, a fluoride sensing riboswitch, was observed to fold into identical tertiary structures, with or without its ligand F^−^, yet triggered gene activation only upon recognition of F^−^ in a narrow concentration range (Zhao et al., 2017). NMR spectroscopy revealed that in the absence of F^−^, the aptamer forms a transitory state of short lifetime (somewhat confusingly termed “excited state” when only thermally-activated sampling is required), which unlocks a linchpin-gated structure that promotes transcription termination. The presence of F^−^ stabilizes the gated conformation toward a functional response within a narrow range of ligand concentrations over a wide range of transcription rates (Zhao et al., 2017). More generally, this example showcases how full refolding of a riboswitch between two alternate secondary structures—which would be slow and come with a thermodynamic barrier likely higher than the energetic driving force available from the RNA binding a small ligand—may often be avoided by an intricate coupling of RNA folding with transcription elongation wherein linchpin events gate the partitioning between the alternate structures (Zhao et al., 2017).

The *yybP–ykoY* family of Mn^2+^ ion sensing riboswitches represent another model for the CS mechanism at physiological concentrations of divalent ions (Figures 1, 2) (Suddala et al., 2019; Sung and Nesbitt, 2019). They have been found to upregulate expression of Mn^2+^ homeostasis genes by binding both Mg^2+^ and Mn^2+^ ions in two adjacent metal ion binding pockets occupying a linchpin position that, once occupied, promotes transcription (Guo et al., 2018; Suddala et al., 2019). The cooperative binding of Mg^2+^ and Mn^2+^ is thought to follow a CS mechanism that stabilizes an adjoining helix P1.1, which in turn competes with a terminator stem that disrupts transcription (Figures 1, 2). Structural studies demonstrate the riboswitch pre-arranging a four-way junction in the presence of millimolar concentrations of Mg^2+^ such that two of the helical arms become transiently juxtaposed, allowing for Mn^2+^ to be captured to turn the riboswitch “on” (Frieda and Block, 2012; Saba et al., 2019) (Figures 1, 2). More broadly, the hierarchy of folding events can be perturbed in riboswitches by the presence of non-cognate ligands and by mutations in the ligand binding aptamer domain. This enables integration over competing metabolic signals and sequence evolution for functional adaptation. Any such perturbation must take effect on the timescale of transcription, emphasizing the kinetic role of ligand and RNA sequence specificity around the aptamer region (Price et al., 2015; Suddala et al., 2015, 2019).

### Correlating the Timescales of Ligand Binding to RNA Folding

Modern biophysical techniques, such as single-molecule fluorescence resonance energy transfer (smFRET) and optical tweezers, have quickly become essential tools for monitoring ligand-dependent structural changes in the aptamer region (Figure 1B) (Savinov et al., 2014). smFRET in particular has been widely used to probe conformational changes in riboswitches at varying concentrations of ligand to correlate ligand binding with RNA folding (Savinov et al., 2014; Suddala and Walter, 2014; Ray et al., 2019). In other cases, such as *in vitro* evolved aptamers with more open binding pockets, the ligand itself can be labeled to monitor its binding to single RNA molecules (Elenko et al., 2009).

In most reports, however, indirect changes in RNA folding and unfolding dynamics are used to probe the mechanism of the aptamer-ligand interaction. For example, one of the smallest riboswitches, the class I preQ_1_-sensing riboswitch follows the two ligand binding mechanisms of IF and CS dependent on the ligand and metabolite conditions as well as specific sequence adaptations found in various bacteria (Suddala et al., 2013, 2015). The transcriptional preQ_1_ riboswitch from *Bacillus subtillis* (*Bsu*) has been observed to favor a CS pathway where the ligand primarily binds to a pre-selected conformation of the aptamer (Suddala et al., 2015). However, the ligand recognition mechanism is fluid, as the same riboswitch can adopt instead the IF mechanism at low metabolite and Mg^2+^ concentrations (Suddala et al., 2015). This dependence of the folding pathway on the relative timescales of ligand binding and conformational dynamics of the aptamer can be identified as a kinetic coupling mechanism occurring early in the decision tree of gene regulation (Figure 1B). Similar kinetic control mechanisms of ligand recognition by the aptamer have been observed to be operational in multiple other riboswitches (Manz et al., 2017; Rode et al., 2018; McCluskey et al., 2019; Sung and Nesbitt, 2019).

### Correlating the Timescales of Riboswitch Folding, Transcription, and Gene Expression

During bacterial transcription, both the kinetics of ligand binding and the speed of RNA transcription determine the functionality of the riboswitch beyond the ligand binding to its aptamer (Wickiser et al., 2005). The coupling observed for the rates of transcription elongation and RNA folding as it emerges from the RNAP exit tunnel in 5′-to-3′ direction highlights the importance of studying riboswitches in the context of the transcription elongation complex. In fact, it has been demonstrated in other RNA folding systems (such as bacterial ribosome biogenesis) that the co-transcriptional directionality of its folding influences an RNA's interactions with known binding partners (Duss et al., 2019; Rodgers and Woodson, 2019). Transcriptional regulatory events, such as pausing, are crucial to the balance between RNA folding and the speed of additional RNA sequence emerging in the wake of RNAP (Saba et al., 2019). Studies have shown how sequence-specific pausing allows the nascent RNA to reach an equilibrium of folded states that then can be further stabilized by RNA binding molecules (Watters et al., 2016; Widom et al., 2018; Rodgers and Woodson, 2019). Depending on cellular conditions, transcription factors such as NusA and NusG are found to stabilize and disrupt transcriptional pausing, respectively (Yakhnin et al., 2016; Guo et al., 2018; Kang et al., 2019). During transcription elongation, a cascade of faster events including RNA folding, ligand binding, and interactions of the RNA with RNAP, together with variations of transcription speed over time, integrate over the biological state of the cell to govern downstream gene regulation (Figure 1B). In light of the reversibility of many, and irreversibility of some, of these steps, conformational and kinetic proofreading becomes possible, adding critical layers of control over the ultimate gene expression outcome (Walter, 2019).

Several recent studies have highlighted details of the co-transcriptional nature of riboswitch folding. High-resolution optical tweezers and single-molecule force spectroscopy approaches showed that a co-transcriptionally folded adenine riboswitch undergoes transcription readthrough predominantly in the presence of adenine, while its absence leads to transcription termination (Frieda and Block, 2012). smFRET assays further demonstrated kinetic control of co-transcriptional folding of a thiamine pyrophosphate (TPP) riboswitch (Uhm et al., 2018). The isolated riboswitch aptamer was observed to fold into a translation “off” conformation independently of its TPP ligand. By contrast, transcriptional pausing allows the riboswitch to rearrange into an “on” conformation in the absence of TPP, while ligand binding steers the nascent RNA into the “off” conformation to downregulate gene expression. This work illustrated that only a brief time window between transcriptional pausing and ligand binding determines the fate of downstream gene expression (Uhm et al., 2018). In case of a F^−^-sensing riboswitch, co-transcriptional *S*elective 2′-*H*ydroxyl *A*cylation analyzed by *P*rimer *E*xtension (SHAPE)-seq revealed that the riboswitch is controlled by the kinetics of co-transcriptional folding, which drives the RNA into a short-lived folded state, even in the absence of F^−^. Binding of ligand favors a kinetically trapped, stably folded state, which delays the nucleation of the terminator hairpin until RNAP has escaped the terminator poly(U) sequence to continue transcription (Watters et al., 2016).

To study the physical interaction between a riboswitch and RNAP, together with the role of a consensus pause sequence on co-transcriptional folding, Widom et al. (2018) performed smFRET, biochemical transcription assays, and molecular dynamics simulations on the paused elongation complex of the class III *que* pause featuring the preQ_1_ riboswitch. This study demonstrated that, on the time scale of transcription, pausing allows the RNAP to slow down and the riboswitch aptamer to sense ligand. This ultimately stabilizes a fully folded RNA pseudoknot conformation that releases the paused RNAP. Additionally, transcription elongation rates likely play a role in riboswitch folding. Early studies in *E. coli* indicated that changes in transcription elongation rate disrupt gene expression and cell growth (Lewicki et al., 1993; Scull and Schneider, 2019). This phenomenon also exists in eukaryotes, where an alteration in the elongation rate of RNAP I disrupts ribosome biogenesis (Schneider et al., 2006, 2007), and a change in RNAP II transcription speed disrupts mRNA splicing (Brzyzek and Swiezewski, 2015), suggesting the universality of such layers of gene expression control. Changes in transcription elongation rate are modulated *in vivo* by covalent modification of the polymerase itself (Fath et al., 2001, 2004), as well as through positive and negative transcription elongation factors, such as NusA and NusG in bacteria (Herbert et al., 2010; Zhou et al., 2011). Future studies on the relationship between transcription elongation rate and riboswitch folding will likely discover additional pause-independent transcriptional regulatory mechanisms. Importantly, future studies should further probe riboswitch folding in the context of RNAP elongation rate, both in the presence and absence of the transcription termination machinery—as both RNA folding and transcription termination could potentially be influenced by RNAP elongation rate.

## Conclusions and Perspectives

Since the discovery of riboswitches in 2002, nearly 20 years ago, methods have dramatically progressed from structural to kinetic studies. These new Technologies are paving the way for a comprehensive understanding of the underlying dynamics under a broad range of conditions and timescales. The structural organization of the aptamer domain and expression platform in the absence or presence of ligand remains critical to a foundational understanding of riboswitch function. Established methods including X-ray crystallography together with more recent advances in high-resolution cryo-EM have provided snapshots of RNA structures that have aided in identifying ligand binding sites and RNA structures (Garst and Batey, 2009; Frank, 2017; Zhang et al., 2019).

Traditional techniques like in-line probing, dimethyl sulfate (DMS) footprinting and SHAPE have allowed for monitoring of structural changes upon addition of a ligand (Soukup and Breaker, 1999; Winkler et al., 2003). Intracellular footprinting by DMS and SHAPE-seq, the latter of which was shown to be able to incorporate selection for active elongation complexes into the original SHAPE protocol (Takahashi et al., 2016; Mitchell et al., 2019), indicate global and some local conformational changes in riboswitches. However, as riboswitches fold asynchronously, smaller local changes may be missed by population averaging (Chauvier et al., 2019; Ray et al., 2019). Advances in single molecule methods such as smFRET and SiM-KARTS have further facilitated our understanding of riboswitches and their mechanisms with the ability to kinetically probe both local and global dynamics and obtain folding and unfolding rate constants (Figure 1B) (Chauvier et al., 2019; Ray et al., 2019). Complementary force spectroscopy experiments have enabled real-time mapping of secondary structure dynamics under perturbation, utilizing magnetic or optical tweezers (Figure 1B) (Frieda and Block, 2012; Tomko and Galburt, 2019). These methods have been instrumental for our understanding of riboswitch dynamics; however, recent advances in the field are increasingly shifting to allow for the study of riboswitches under more biologically relevant conditions.

The overall goal of the field of riboswitch biology remains the same: to understand the mechanisms by which riboswitches bind ligands to transduce a signal through conformational changes in the expression platform for ultimate control of gene expression. Recent studies have focused on the importance of co-transcriptional folding and how the elongation complex affects riboswitch mechanism, and vice versa (Watters et al., 2016; Ray et al., 2019; Strobel et al., 2019). Transcription rates can range from 10 to 25 nucleotides per second, and the RNA immediately starts folding directly after exiting RNAP, leaving only a short time window for riboswitches to sample alternative folding pathways in service of gene regulation (Dangkulwanich et al., 2014). Co-transcriptional studies are beginning to highlight the importance of the context of the transcription machinery and the critical role that integration of timescales plays in the mechanisms of gene regulation (Figure 1B) (Watters et al., 2016; Ray et al., 2019; Strobel et al., 2019). Conversely, the discovery of the functional importance of co-transcriptional riboswitch folding has driven the development of techniques such as co-transcriptional SHAPE-seq and artificial RNA elongation complex assembly (Watters et al., 2016; Strobel et al., 2019). Co-translational SHAPE-seq, in turn, has enabled high-throughput structural probing of RNAP complexes halted *in vitro* at various transcript lengths to obtain single-nucleotide resolution of the nascent RNA (Watters et al., 2016). To truly understand how ligand binding by a riboswitch couples to gene expression, future studies must increasingly monitor these events in concert and acknowledge that RNA acts as an active effector of gene regulation rather than a passive output. Integrating riboswitches into their biologically relevant contexts will require directly monitoring single molecules at a broad range of timescales and including a plethora of external cofactors that may influence folding mechanisms during and after transcription.

Technical developments on the horizon will include single molecule assays that monitor elongating RNAP complexes using total internal reflection fluorescence (TIRF) and zero-mode waveguide (ZWM) microscopy at increasingly higher throughput of individual molecules while maintaining high sensitivity (Duss et al., 2018, 2019). Protein Induced Fluorescence Enhancement (PIFE) has emerged as a way to measure transcription rates and, when coupled with FRET or fluorescent probes to monitor ligand binding and dynamics of the transcript, enables the measurement of real-time kinetics during transcription (Duss et al., 2018, 2019; Rodgers and Woodson, 2019). There is also potential for three- or four-color smFRET to monitor multiple dynamic interactions simultaneously within a single riboswitch (Lee et al., 2010). These developments will allow for kinetic measurements under varying conditions, including at RNAP pause sites and in the presence of transcription factors, divalent ions, and other intracellular factors, without the need for synchronizing individual molecules by sudden perturbation (Suddala et al., 2015; Gabizon et al., 2018; Widom et al., 2018). Extending these cutting-edge techniques to riboswitches will beget a deeper understanding of how RNA can efficiently integrate environmental cues over broad timescales to affect bacterial gene expression control and survival, with the promise of boosting our ability to suppress bacterial infections with antibiotics.

## Author Contributions

CS, SD, RR, and NW wrote the manuscript. CS and SD produced the figures. All authors contributed to the article and approved the submitted version.

## Conflict of Interest

The authors declare that the research was conducted in the absence of any commercial or financial relationships that could be construed as a potential conflict of interest.
